# Pregnancy loss in women with systemic lupus erythematosus: Grounded Theory

**DOI:** 10.1590/0034-7167-2023-0225

**Published:** 2024-05-03

**Authors:** Rebeca Rosa de Souza, Mayckel da Silva Barreto, Elen Ferraz Teston, Maria Aparecida Salci, Viviane Cazetta de Lima Vieira, Sonia Silva Marcon

**Affiliations:** I Universidade Estadual do Paraná. Paranavaí, Paraná, Brazil; II Universidade Estadual de Maringá. Maringá, Paraná, Brazil; III Universidade Federal do Mato Grosso do Sul. Campo Grande, Mato Grosso do Sul, Brazil

**Keywords:** Systemic Lupus Erythematosus, Abortion, Spontaneous, Pregnancy, High-Risk, Grounded Theory, Symbolic Interactionism, Lupus Eritematoso Sistémico, Aborto Espontáneo, Embarazo de Alto Riesgo, Teoría Fundamentada, Interaccionismo Simbólico, Lúpus Eritematoso Sistêmico, Aborto Espontâneo, Gravidez de Alto Risco, Teoria Fundamentada, Interacionismo Simbólico

## Abstract

**Objective:**

to learn the meanings attributed to pregnancy loss by women with Lupus.

**Method:**

qualitative research, based on Symbolic Interactionism and Grounded Theory. Data collection took place between January and August 2022 through in-depth interviews. Data analysis went through the stages of initial and focused coding.

**Results:**

seventeen women participated. The central phenomenon “The climb to motherhood: falls and overcoming” was constructed, consisting of three categories: “Falling to the ground during the climb: the experience of pregnancy loss”; “Getting up and following the path: new attempts to conceive”; and “Remembering the journey: meanings attributed to pregnancy losses”.

**Final considerations:**

experiencing pregnancy is, analogously, like climbing a mountain, where obstacles need to be overcome to reach the summit. The experience of pregnancy loss is seen as complex, especially when there is fragility in healthcare and a lack of awareness regarding feelings of loss and grief.

## RESUMO

**Objetivo:**

apreender os significados atribuídos à perda gestacional por mulheres com lúpus.

**Método:**

pesquisa qualitativa, pautada no Interacionismo Simbólico e na Teoria Fundamentada nos Dados. A coleta de dados ocorreu entre janeiro e agosto de 2022, mediante entrevistas em profundidade. A análise de dados percorreu as etapas de codificação inicial e focalizada.

**Resultados:**

participaram 17 mulheres. Construiu-se o fenômeno central “A escalada da maternidade: quedas e superações”, constituído por três categorias: “Caindo ao chão durante a subida: a vivência da perda gestacional”; “Levantando e seguindo o caminho: novas tentativas de gestar”; e “Rememorando o percurso: significados atribuídos às perdas gestacionais”.

**Considerações finais:**

vivenciar a gestação é, analogicamente, como escalar uma montanha, onde obstáculos precisam ser vencidos até o alcance do cume. A vivência de perdas gestacionais é significada como complexa, em especial quando há fragilidade na assistência à saúde e falta de sensibilização diante dos sentimentos de perda e luto.

## RESUMEN

**Objetivo:**

comprender los significados atribuidos a la pérdida del embarazo por mujeres con Lupus**. Método:** investigación cualitativa, basada en el Interaccionismo Simbólico y la Teoría Fundamentada en Datos. La recolección de datos se realizó entre enero y agosto de 2022, mediante entrevistas en profundidad. El análisis de datos pasó por las etapas de codificación inicial y focalizada.

**Resultados:**

participaron 17 mujeres. Se construyó el fenómeno central “La subida a la maternidad: caídas y superación”, conformado por tres categorías: “Caer al suelo durante la subida: la experiencia de la pérdida del embarazo”; “Levantarse y seguir el camino: nuevos intentos de concebir”; y “Recordando el viaje: significados atribuidos a las pérdidas de embarazos”.

**Consideraciones finales:**

vivir el embarazo es, análogamente, como escalar una montaña, donde es necesario superar obstáculos para llegar a la cima. La experiencia de la pérdida del embarazo se considera compleja, especialmente cuando hay fragilidad en la atención de salud y falta de conciencia sobre los sentimientos de pérdida y duelo.

## INTRODUCTION

Pregnancy constitutes an essential physiological process for human reproduction, and is characterized by hormonal, physical, psychological, family and social changes^(1-2)^. In the presence of autoimmune pathologies, such as systemic lupus erythematosus (SLE), pregnancy becomes high risk and may compromise maternal and fetal health^(3)^.

Of unknown etiology, SLE can affect multiple organs such as skin, joints, heart, lungs, kidneys and brain, in addition to causing dysregulation in the immune system^(3)^. Its incidence worldwide is approximately 1 to 22 cases for every 100,000 inhabitants per year, and the prevalence can vary from 7 to 160 cases for every 100,000 people^(4)^. In Brazil, an estimated incidence of around 8.7 cases per 100,000 people per year, occurring more frequently among women of reproductive age^(4)^. Among pregnant women, the incidence varies from 1: 660 to 1: 2,952, leading to significant maternal and fetal morbidity^(5)^. The combination of SLE and pregnancy is related to a high risk of complications, including pregnancy losses^(6)^.

Pregnancy is often perceived as an important moment in a woman’s life, in which the idealization of the dream of motherhood is experienced progressively, and its abrupt interruption, associated with the resulting losses, can trigger significant physical and psychological suffering, in addition to feelings of incapacity, denial, guilt, frustration and fear of future pregnancies^(7)^.

At the same time that SLE brings complications to pregnancy, it can exacerbate the manifestations of the disease^(8-10)^. In the presence of antiphospholipid syndrome (APS) - a thromboembolistic condition that favors the occurrence of venous and arterial thrombosis^(11-12)^ -, the risk of miscarriage is greater^(11-13)^. During pregnancy in women with SLE, the incidence of APS is approximately 40% and, without adequate treatment, approximately half of cases progress to pregnancy loss^(12)^. Likewise, such risks are elevated in the presence of anti-SSA/Ro or anti-SSB/La antibodies^(11-13)^, especially when pregnancy occurs without reproductive planning and without treatment/monitoring of risk factors.

In the presence of a chronic autoimmune condition such as SLE, in common sense, motherhood is often perceived as impossible or very difficult to achieve. This contributes to pregnancy being postponed or even excluded from the future plans of women and their families. In this scenario, when women become pregnant and, consequently, suffer a miscarriage, the attribution of meanings to this period can have negative impacts, especially in the case of those women who experience the pain of grief and loss silently. It is also noteworthy that, when the loss occurs at the beginning of pregnancy, grief is socially neglected by family, friends and healthcare professionals^(7)^.

Previous studies have shown that experiencing pregnancy loss, whether single or recurrent, contributes to the occurrence of psychosocial illnesses, as women experience feelings such as hopelessness, frustration and guilt^(14-15)^. In this regard, healthcare needs to consider the subjectivity of each process, and it is necessary to seek to understand and offer care based on the uniqueness and needs of each woman.

In autoimmune conditions such as SLE, obstetric complications and pregnancy losses can be avoided, as long as women are prepared for pregnancy in a timely manner. To this end, she needs to receive guidance regarding the importance of planning the pregnancy so that it occurs when the disease has been in remission for at least six months after preconception^(3)^. Such a measure can reduce the risk of pregnancy loss and help women with SLE experience a calmer and safer pregnancy. Therefore, learning the meanings attributed by women with SLE in relation to the experience of pregnancy loss may favor the planning of healthcare offered to this public, especially with regard to the recognition of the subjectivities necessary to cope.

## OBJECTIVE

To learn the meanings attributed to pregnancy loss by women with lupus.

## METHODS

### Ethical aspects

This is a study developed in accordance with the guidelines of Resolution 466/12 of the Brazilian National Health Council and in accordance with the guidelines for research procedures in a virtual environment of the Brazilian National Research Ethics Commission (CONEP - *Comissão Nacional de Ética em Pesquisa*). Project approved by the Human Research Ethics Committee of the signatory institution. In the description of this research report, the COnsolidated criteria for REporting Qualitative research (COREQ) guidelines were respected^(16)^.

All participants signed the Informed Consent Form (ICF), made available via Google Forms, and validated this consent verbally before the start of the interview. To guarantee their anonymity, the extracts from speeches were identified by the letter P (participant) and an Arabic number, applied according to the interviews (e.g., P1).

### Theoretical-methodological framework

Symbolic Interactionism (SI) was used as a theoretical framework because it allows understanding human action based on the way individuals act and behave when faced with an object or phenomenon. This framework presupposes that meaning is created from experience, through interaction with the object and with the individual that defines it. Thus, the value of a given experience and the action triggered in relation to it arise from the meaning that the person attributes to it. It can be said that SI studies individual and group behavior, dealing with the experiential issue, i.e., how people define events or reality and how they act in relation to their definitions or beliefs^(17-18)^.

When experiencing a pregnancy with SLE, the meaning of this process is defined through women’s personal interaction with the phenomenon and their social interaction with the people around them. Such meaning is created, recreated and redefined according to the way this phenomenon presents itself in their personal and social life, with their behavior, acts and meanings being created from their own concepts and the behavior, attitudes and perspectives of others in relation to their condition.

As a methodological framework, Grounded Theory (GT) was adopted from a constructivist perspective. This approach recognizes that substantive theory is constructed from the collection and analysis of concomitant data and that it involves the researcher’s active interpretation of the phenomenon under investigation^(19)^. However, GT stands out as a support for qualitative research, as it is an investigation method that aims to investigate, understand and create a theory based on the development of a phenomenon^(19)^, allowing the researcher to carry out an in-depth investigation, whether with individuals or groups, favoring the construction of significant scientific evidence that favors the interpretation of a social reality comprehensively.

### Study design and place

This is a qualitative study, in which data were collected remotely. The recruitment platform was Facebook^®^, specifically in the group “*Lúpus Brasil - o desabafo*”. Created in 2014, the group has more than 30 thousand members, of which around 90% are women. The group’s objectives are to exchange information and experiences with the disease, and topics such as pregnancy and abortion are frequently discussed. It is noteworthy that the main researcher has been a member of this group since 2018, when she developed a study for her master’s degree in nursing with the aim of learning the perception of people with SLE about living with the disease. Over the years, she has interacted with other members by exchanging messages and posts about lupus and pregnancy.

### Data collection

Data were collected between January and August 2022, using individual, in-depth interviews, carried out via WhatsApp^®^ and Messenger^®^ video calls, with audio and video recorded after authorization. The average duration time was 75 minutes. All interviews, as well as concomitant data analysis, were carried out by the main researcher, a doctoral student in nursing, with experience in collecting and analyzing qualitative data.

A total of 17 women participated in the study, divided into three sample groups. The first group was created intentionally and included women with SLE who had experienced pregnancy loss. The research invitation was made through a public post, with the researcher’s presentation, research objective and remote data collection technique. The women expressed interest in participating in the study through a response to the post and also via direct, at which time the day and time for the video call were scheduled according to participants’ availability and preference. At this time, there was a more detailed presentation by the researcher and participant, clarifying the reasons for the research and the objective of investigation.

Women aged 18 years or over, diagnosed with SLE, having experienced at least one pregnancy loss and having access to the internet were included. In turn, women who presented communication difficulties, such as muteness or deafness, would be excluded, but the criterion was not used. The first sample group consisted of seven women, with no refusals to participate or withdrawals.

Through concomitant data collection and analysis, it was evidenced that, in addition to the diagnosis of SLE, some women had APS, and, when this clinical condition was present, the number of pregnancy losses was more recurrent as well as the perception and meaning attributed by women to the experience were different from those who only had SLE as a chronic condition. Therefore, the following hypothesis was raised: suffering pregnancy losses with two autoimmune conditions that significantly impact gestational outcomes changes the way women perceive, act and behave in the face of the loss. To confirm or not this hypothesis, theoretical sampling was extended and the second sample group was constituted.

This consisted of six women who were diagnosed with SLE and APS. Recruitment also took place remotely in the private group “*Lúpus Brasil - o desabafo*”. All participants who expressed interest in constituting the second sample group participated in the study, with no dropouts. The inclusion criteria were: having developed APS during pregnancy and having experienced at least one miscarriage of this combination. In the concomitant analysis process, it was evident that the meaning attributed to pregnancy loss was even more significant and intense when women experienced recurrent losses, raising the following hypothesis: experiencing repeated pregnancy losses changes the way women perceive and mean abortion. Thus, the theoretical sampling was extended.

The third sample group consisted of four women with SLE and APS who experienced repeated pregnancy losses (at least two losses). The following inclusion criteria were added for recruitment: having been diagnosed with SLE and APS and having experienced repeated pregnancy losses, i.e., more than one consecutive pregnancy loss. These were also recruited on the online platform. There were no refusals or withdrawals.

As guiding questions, the following were applied: sample group I: tell me about the pregnancy loss(es) you suffered as a result of SLE; sample group II: tell me about the pregnancy loss(es) you suffered as a result of SLE and APS; sample group III: tell me about the repeat pregnancy losses you suffered in SLE and APS pregnancies. Whenever necessary, supporting questions were used, for example: how did you feel at that moment? Tell me more about this. What meanings do you attribute to this experience? The recruitment of new participants occurred until the data analysis did not identify new information, i.e., it did not provide more depth to the categories found and the objective of the study was achieved. The theoretical saturation process was used according to the recommendations set out in the adopted methodological framework^(17)^. There was no need for repeated interviews.

### Data analysis

Data were organized and analyzed using the MAXQDA plus 2020 software. For the analysis process, the interviews were transcribed (not returned to participants for comments and/or corrections due to the unavailability of time to complete the research), and the texts are studied and data is approached, highlighting the most significant and similar ones, always in comparison with each other and with the memos and diagrams constructed throughout the process, and reconstructed based on evidence.

The initial coding process occurred word by word, line by line and incident by incident, with codes also being identified *in vivo*. For the coding process, gerund was used, totaling 4,441 initial codes. Subsequently, the initial codes were subjected to focused coding, in which the most significant and frequent ones were compared to each other, allowing the coding of more selective and conceptual codes, which gave rise to the categories.

## RESULTS

The 17 women in the study were between 24 and 46 years old, with menarche between 10 and 15 years old and beginning of sexual activity between 13 and 20 years old. The time since SLE diagnosis ranged from two to 16 years. All of them had already experienced at least two pregnancies (maximum of six) and one pregnancy loss (maximum of five). All participants managed to have children. The main comorbidities associated with SLE reported were APS (17), fibromyalgia (3), hypothyroidism (1) and Raynaud’s Syndrome (1). Women from the states of Paraná, São Paulo, Rio de Janeiro, Mato Grosso do Sul, Santa Catarina, Minas Gerais, Goiás, Paraíba, Espírito Santo and the Federal District participated. One of the participants lived in Sweden.

**Figure 1:**
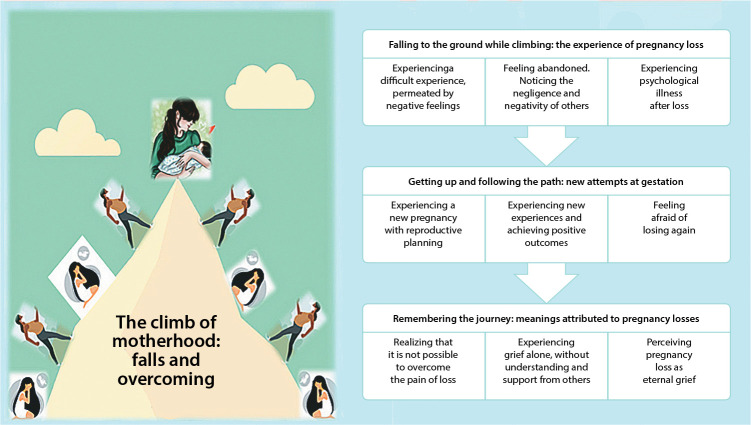
Diagram of the graphic representation of the central phenomenon, categories and subcategories of the study

From the analysis of interviews, the central phenomenon “The climb of motherhood: falls and overcoming” was constructed, consisting of three categories: “Falling to the ground during the climb: the experience of pregnancy loss”; “Getting up and following the path: new attempts at pregnancy”; and “Remembering the journey: meanings attributed to pregnancy losses”. Figure 1 presents the diagrammatic illustration of the central phenomenon, interrelated categories and subcategories. It was possible to identify that the experience of pregnancy with SLE and pregnancy losses are, analogically, meant as climbing a mountain. In this process, dealing with obstacles leads to falls (pregnancy loss). The obstacles refer to symbolic interaction with others, permeated by the fragility of healthcare, lack of family support and suffering due to grief, sometimes understood as eternal, which need to be worked on and overcome until the experience of a pregnancy that culminates in the birth of a child. Such an achievement (having a child) is signified as reaching the top of the mountain. However, reaching the top of the mountain does not mean forgetting the path traveled and the pain experienced.

### Falling to the ground during the climb: the experience of pregnancy loss

Analogously, in this study, pregnancy loss was compared to a fall while climbing a mountain. Experiencing pregnancy loss by women with SLE is, in most cases, associated with their underlying diseases, i.e., women, based on social and symbolic interaction with the world around them, give new meaning to the interrupted pregnancy as a consequence of SLE and/or APS diagnoses.

*The first two losses I didn’t know were due to lupus, the last two yes, everything happened because of lupus.* (P13)

*APS does this during pregnancy. If you don’t take care of it correctly, you lose, that’s what happened to me. The lupus was in remission, but APS was not.* (P5)

*If I didn’t have this disease, I wouldn’t have lost my children.* (P15)

During the pregnancy loss process, women need to be assisted by healthcare professionals. Regarding the assistance received, they report that professionals’ perception of the deliberate association between abortion in SLE constitutes a normal and, to a certain extent, expected occurrence, which contributes to the experience being seen as difficult and marked by feelings of denial, revolt and incapacity.

*I got tired of hearing from professionals that having an abortion was the most normal thing in the world, that women with lupus cannot get pregnant, cannot carry a child. This always made me very angry, sad, that’s why to this day I still don’t accept that I have this disease.* (P3)

*When I discovered I had lupus, the doctor told me to have a tubal ligation so as not to run the risk of getting pregnant, because both I and the baby would die. I spent my life believing this, feeling guilty, incapable.* (P5)

Needing hospital admission after the occurrence of pregnancy loss is also seen as a complicating factor for the experience to be permeated by suffering. This occurs, in particular, when women are admitted in the same ward where women who have just had their children are admitted to hospital. At this time, they need to interact with other women and their newborn children, in addition to symbols that refer to new motherhood such as crying and breastfeeding. This situation is understood by women as negligence and lack of sensitivity by healthcare professionals, who amplify suffering.

*I stayed in the maternity ward listening to babies cry all the time, along with other women breastfeeding with their children in their arms, and me without. I found it inhumane, I suffered a lot.* (P3)

*I was admitted to the same room as a woman who had just had a baby, and I was there waiting to have mine removed, to do the scraping. That finished me off. I even asked to be changed rooms, but no one did anything.* (P4)

Even more negative feelings are perceived when a woman who suffered a pregnancy loss also loses the right to be accompanied by a family member during the period of hospital admission, not having her right to be a parturient recognized.

*I went in alone, my husband was at the reception, I only went to see him after the surgery, in the room, and he couldn’t stay with me. I was the only one without a companion, I didn’t have a baby, I didn’t need a companion. And I lost it after Christmas, it was very difficult, painful, being alone, in pain, sad, being treated in any way by professionals. Inhumane, right?* (P3)

*I was alone the entire hospital stay, before the scraping and after, they didn’t let my husband stay with me, nor my mother, anyone.* (P4)

The sadness and suffering experienced in the face of pregnancy loss persists after the hospital admission period. Women experience the pain of grief, which is neglected by other people, who do not understand nor are they sensitive to pregnancy loss. Negative reports, such as normalization of loss, “soon you get pregnant again and you have to accept it”, are frequent and bring even more sadness to women who have suffered a pregnancy loss.

*Losing a child is the worst thing that can happen in a woman’s life. My world fell apart, because it was a dream, I was so happy and suddenly I lost it. And no one understands the pain we feel, they think it’s normal and exaggerated for us to suffer, but only those who go through it are able to understand.* (P2)

*People don’t understand our suffering, they don’t seem to believe it, they keep saying things we don’t want to hear. Like: you have to accept it, it was better this way, God knows all things, soon you get pregnant again, and that bothers you, it’s not nice to hear.* (P7)

Faced with the suffering experienced by losses, as well as the lack of social support, some women need to seek professional support, when they realize that they would not be able to go through the grieving process alone.

*I did many years of therapy which helped me a lot. I wouldn’t be able to recover alone. I needed help to understand and accept everything that had happened to me.* (P7)

*I needed psychological treatment after the losses. It was very difficult for me to overcome. It was a dream to be a mother, then I really felt bad.* (P10)

The results of this category showed that, although the pregnancy loss of women with SLE is associated with the underlying disease, sometimes, when naturalized by professionals, having their rights violated, feeling without social support and lack of sensitivity from the team during hospital admission, generate feelings and meanings loaded with negativity.

### Getting up and following the path: new attempts at gestation

After experiencing the occurrence and/or recurrence of pregnancy loss, some women signify and associate such losses with their underlying diseases, in a process of seeking to understand pregnancy within the context of autoimmune diseases (SLE and APS). In the meantime, women, when interacting with healthcare professionals and seeking further clarification about their conditions, come to understand the need and relevance of adequately planning their pregnancy in advance. Analogically, it is as if women got up after falling, planned the climb better and continued trying to climb the mountain.

*After a long time, I managed to learn about lupus. I studied a lot about it, and only after that, I was able to understand that I lost my baby because I didn’t plan the pregnancy beforehand. I got pregnant taking strong medication that wasn’t recommended, so I lost it.* (P4)

*It was only after the two miscarriages that I was able to understand why I lost babies. The rheumatologist I’m seeing today explained to me that when I got pregnant my lupus was active and that was possibly why I miscarried. He also diagnosed APS, which may have contributed to lupus in miscarriages.* (P9)

Based on this process of social interaction and the search for understanding about the importance of reproductive planning in a pregnancy with SLE, some women are able to experience a new gestational process, in a planned way and with different care from previous experiences, seeking to obtain more positive gestational outcomes.

*In the last pregnancy, I did all the planning together with the rheumatologist and then with the obstetrician, and it worked, I was able to have my son.* (P17)

*In my fourth pregnancy, I used injections for APS. The lupus was sleeping, I was barely taking any more medication, I was out of risk, and then it worked.* (P2)

However, even when experiencing a new gestational experience, the diseases symbolically and historically marked in women’s minds awaken feelings of fear in the face of the real or imagined possibility of losing something that is not yet something concrete. This causes women to feel a strong sense of insecurity in the face of the possibility of a repeated negative pregnancy outcome, which only ends when a child is born.

*I was very afraid, mainly, of losing again. We feel insecure. I only really believed it when I held it in my arms.* (P2)

*I think fear and insecurity were the biggest feelings.* (P16)

Experiencing the process of gestation with SLE and APS is relational and permeated by different interactions, especially with healthcare professionals. Regarding the healthcare received, women realize that, when they are supported by the health team, pregnancy is experienced with more security and tranquility, in the same way that the end of the gestational process is more favorable and positive.

*Certainly, the care of doctors and the correct prenatal care were essential. I felt safer, less afraid of losing again, and that helped a lot.* (P11)

*I followed the entire pregnancy with the medical team until after the birth. Here they make home visits in the postpartum period to see if everything is ok and if the baby is breastfeeding properly. There is a lot of care and attention, I have nothing to say, I was very well looked after.* (P17)

Reaching the realization of the dream of motherhood is meant as an achievement and overcoming, since pregnancy losses were experienced along the way, leading women to experience the pain of grief and the interruption of maternal desire. In this process, feelings, such as disbelief that I could be a mother, are experienced. However, after an arduous process of repeated attempts, surrounded by the ambiguity of hope and suffering, these women persist, manage to get up and try again, thus achieving the dream of motherhood.

*I thought I would never be able to have a child and, when I least expected it, it happened. I am very happy to have had this opportunity. I am very grateful to God. I gave it to God, I had a lot of faith and also a lot of strength, because it wasn’t easy.* (P7)

*I went against my family, my husband, the doctors, everyone. I insisted a lot, I suffered too, but I managed.* (P8)

The results show that, based on understanding the importance of planning the pregnancy in advance and gestating with the disease in remission, a new pregnancy can be experienced and experienced in a lighter way, achieving positive gestational outcomes.

### Remembering the journey: meanings attributed to pregnancy losses

It was identified that women with SLE and APS, in a similar way to climbing a mountain without due preparation, when going through the pregnancy process, experience one or recurrent pregnancy losses (falls during the climb). After learning more about the path to be taken, they prepare themselves and reach the top of the mountain, motherhood. Experiencing this process brings marks and scars that, despite being in the past, continue to be felt in the present time and reverberate in women’s lives. To formulate and rescue these memories, women consider the self-interaction they had with the phenomenon, as well as social interaction, i.e., the way other people perceived and behaved in the face of this suffering.

*Today, my biggest pain is having this disease, because it is what caused this. I still haven’t gotten over the losses, they still hurt me a lot. Even though I have my son here with me, I still grieve for those I lost. This brings me an emptiness, a great sadness. I still feel guilty.* (P2)

*The way people treated me hurt me even more, because I wanted a hug, a word of comfort, a shoulder to lean on, but I didn’t have one, what I got were words of: “Get over it, this will pass”. Every time I heard this, I felt even more incapable. I still feel guilt.* (P7)

Going through the process of loss reflects a complex process, and it is not possible to disentangle the feelings of fear and grief of future pregnancies. In this regard, when experiencing a new gestational process, women carry memories of previous experiences.

*For me, I have a son in heaven, even if no one understands or believes in my grief, but he exists and I live him every day of my life.* (P1)

Through this, loss is symbolized as a pain that cannot be overcome. Even though time passes and new opportunities are experienced, women who have experienced the loss of a child, whether intrauterine or not, carry over time the marks and scars of early pregnancy terminations, in which feelings of guilt, sadness and pain are experienced.

*We don’t overcome the loss, we learn to deal with the pain, with the sadness, but we always remember, we feel guilt, sadness, pain in the heart, “but” we learn to deal with the pain.* (P5)

*Losing a child is the worst pain a person can feel in their life. There’s no way to forget, how to overcome, it’s a wound in the chest that will never close.* (P14)

However, in addition to remembering the negative experience previously experienced, pregnancy loss is also perceived and symbolized as an eternal grief, since women do not accept and do not overcome the early loss of their children, they also feel alone in this process, referring to a grief that has been experienced and is lived alone over time.

*I will never forget, I will never get over. It’s my grief. A grief that I live alone, a grief that I carry in my heart, the grief of my baby that I lost.* (P13)

*There is no way to explain the pain of losing a child, it is a grief that one lives alone forever. No one is capable of understanding a mother’s pain, people don’t value our pain, they don’t recognize it, they think it’s nonsense, that it’s not possible to love a child you haven’t held in your arms. Only a mother who lost her son can understand this.* (P16)

The reports in this category demonstrate that pregnancy loss is perceived as a complex experience that negatively impacts the meanings that women attribute to pregnancy, which is symbolized as something that cannot be overcome and an eternal grief.

## DISCUSSION

The results demonstrate that the meanings attributed to pregnancy loss are constructed and given new meanings based on the lived experience. Self-interaction is the phenomenon, and social interaction is responsible for the way women behave, perceive and signify the process experienced. Such perspectives are in line with the assumptions established by the adopted theoretical framework, which predicts that meanings are constructed based on the way each individual lives the experience, with personal interaction and social interaction being the pillars for the construction of these meanings^(17-18)^.

Pregnancy in women with SLE is, in most cases, a desired process and also a very feared one, especially due to the risks to which they are exposed. Among the main complications, the occurrence of pregnancy losses stands out, favoring the idea that pregnancy with SLE is complex and, in some situations, contraindicated^(8-9)^. A retrospective cohort study of 149 pregnancies in 98 women with SLE, carried out in Oman, highlighted the presence of pregnancy loss as one of the main complications, with the occurrence being identified in more than 50% of the population studied^(13)^. Another Colombian cohort study, carried out with 48 pregnant women, identified similar results, concluding that the occurrence of pregnancy losses in pregnancies with SLE represents one of the main complicating factors in this population^(20)^.

A study carried out in Iran with 27 women with SLE identified that feelings of fear, insecurity, hopelessness and uncertainty were frequently experienced by them^(21)^. The abrupt experience of a pregnancy loss for the first time or on a recurring basis contributes to the attribution of negative meanings. Sometimes, pregnancy, in addition to being desired, is symbolized as a dream^(7,22)^. Therefore, the women in this study associate pregnancy losses with SLE and the presence of APS, leading to the understanding that, due to these diseases, the dream of motherhood was interrupted early. The discussion of this meaning needs to be incorporated into the daily care of women with SLE so that these precepts are demystified through reproductive planning actions.

Feelings of frustration, anger and inability in relation to healthcare were also reported as complicating factors in the gestational process, especially due to the stance of professionals who naturalize the occurrence of abortion in women with SLE or contraindicate pregnancy because they consider that positive pregnancy outcomes are not possible.

However, with greater diagnostic accuracy and access to health services, pregnancy in women with SLE is possible^(10,23-24)^. Experiencing contraindication, as well as hearing discouraging advice from healthcare professionals, results in emotional turbulence and pessimism, which contribute to psychological illness and favor a complex and negative experience^(21,25)^.

Reports from some women show that they were denied the right to a companion because they were not considered pregnant and/or giving birth. However, Federal Law 11,108 of April 7, 2005 guarantees women in labor the right to the presence of a companion during labor, birth and immediate postpartum, in their own or partner network of the Brazilian Health System (SUS *- Sistema Único de Saúde*)^(26)^. In this sense, when entering the hospital environment, whether for curettage or the birth of a live child, every woman must have the right to a companion preserved. The presence of a loved one at this moment contributes to welcoming women and can provide emotional support to face the situation. However, this right is often not known by women and neglected by institutions and healthcare professionals.

In agreement, some research consistently reflects gaps in care for women with pregnancy losses. A systematic review, which included 30 articles from 11 countries, including the United States of America (USA), Australia, the United Kingdom and Brazil, showed that the lack of professionalism in the hospital environment can negatively affect the emotional well-being of parents who have suffered pregnancy loss, especially the mothers who, in addition to the interruption of pregnancy, experience the physical and emotional transformation of woman, pregnant woman, mother transition and loss^(27)^.

The lack of empathy, the recognition and treatment of abortion as something normal and routine, the healthcare professionals’ coldness and the lack of understanding in understanding feelings of grief and loss are also cited as influencing this process^(14)^. The reception offered by healthcare professionals to women after an abortion can influence the way they perceive, act and give meaning to their experience^(14)^.

In this regard, whenever possible, it is important that professionals also pay attention to the place where women with pregnancy losses are admitted to hospital. Reports show that being admitted to the same room and/or environment as women in labor and especially those who have just had their children represents additional suffering for them.

Therefore, avoiding sharing the environment with other women and allowing the presence of a companion during hospital admission for curettage constitutes a form of care and empathy that can help women cope better with this situation, in addition to not feeling alone and helpless. Thus, it is important that, in health institutions, there are protocols that guarantee the humanization of care and respect for women’s rights as parturient women.

In this regard, it is urgent to consider that healthcare centered and valued in the biomedical model tends to be limiting in meeting women’s health needs other than morphofunctional ones. The care model based exclusively on the biological causes feelings of dissatisfaction and distrust with the assistance offered^(28)^.

Non-recognition of pregnancy loss by third parties in the process of social interaction also influences the attribution of meanings. A study shows that silence and the lack of appreciation by other people for sadness and suffering make women feel even more insecure. This, in turn, favors the development of post-loss anxiety and depression, which impacts a future pregnancy^(14)^.

In this sense, social contact with other people, the ways in which they behave in the face of pain and the frustration experienced produce impacts on the construction of meanings, which are modeled and given new meanings based on the “social self”. Both healthcare professionals and family, friends and loved ones are considered significant in the process of adapting to and overcoming pregnancy loss^(14)^. Therefore, this expanded support network is important in the process of coping with pregnancy loss and the feeling of grief, contributing positively to damage reduction and reducing the risks of illness and post-abortion psychosocial disorders^(15)^.

Although the grieving process is perceived and felt differently by people who experience the same type of loss, it needs to be understood as resulting from a social context. People live in society, and this influences feelings and behaviors when faced with the loss of someone. For this reason, the psychological elaboration of grief results from the way a social group thinks about death and behaves towards it^(29)^, which corroborates the assumptions of SI as well as the reports evidenced in this study.

After experiencing an abortion, some women are able to realize the importance of reproductive planning and the need for pregnancy to occur when SLE is in remission. In fact, it is important that the disease is in remission for at least six months^(3)^, because, if conception occurs during the inflammatory activity of the disease, there is a significant increase in “flares” (activation of disease), with inevitable interruption of pregnancy^(30)^.

Reproductive planning is a relevant tool in high-risk pregnancies, especially in autoimmune diseases, favoring the process and positive gestational outcomes^(10,23-24)^. Through this, some women are able to plan and experience a new gestational experience, thus giving new meaning to the “self”, although feelings of fear and insecurity are still present.

Studies show that fear is a very common feeling in the lives of women who have SLE. They are afraid of death, physical and emotional disabilities, pregnancy loss and the possibility of transmitting SLE to their children^(21,31-32)^. Thus, receiving support from healthcare professionals when faced with a new experience stands out as a strategy that influences good gestational outcomes.

It is worth noting that, even with the birth of a child, pregnancy loss continues to be seen as an insurmountable pain and eternal grief. Such feelings are explained in the literature as a common and individual process, and it is not possible to explain or treat it through clinical protocols^(14-15,29)^. Therefore, each case must be studied and valued subjectively, with healthcare being offered according to each need.

### Study limitations

Among the possible limitations of this study, the highlight is that the informants were located through a virtual platform (Facebook^®^) and, therefore, the results of this study are restricted to women participating in the group and subject to contextual influences from this means of communication, and the fact that the interviews were carried out remotely, in addition to participants being selected based on their willingness to share their experiences with the phenomenon under study. Therefore, women with lower communication skills were not considered as potential participants.

### Contributions to nursing and health

There is a need to disseminate information about pregnancy losses in women with SLE among healthcare professionals, people with SLE and the community in general, in order to help them understand this phenomenon, which could favor the process of social interaction of this public, contribute so that feelings of loss and grief can be experienced in a less traumatic way and favor the process of coping and overcoming.

In the health field, such knowledge can contribute to professional training in the face of these demands and favor the development of institutional protocols that permeate the humanization of care for women experiencing pregnancy loss, such as the separation of hospital wards and respect for the companion law. women in labor, whether a child is alive or not. Furthermore, the results reiterate the need to emphasize, during the training process, on expanded care, i.e., that enables healthcare professionals to visualize sick individuals beyond their chronic condition.

## FINAL CONSIDERATIONS

The results showed that experiencing pregnancy with SLE, in the analogy of climbing a mountain, is a journey where obstacles need to be constantly overcome until reaching the much-desired summit (having a child). The presence of obstetric complications, as well as occurrence of pregnancy losses, is more common when there is an absence of reproductive planning. However, even in the face of falls (pregnancy losses), women are able to get up and follow the path, trying the pregnancy process again and reaching the top of the mountain when the pregnancy occurs as planned and with the disease in remission.

Along this path, the experience of pregnancy loss has negative impacts on women’s lives, in particular, on their perceptions about life, their feminine identity and their role in society. Feelings of loss and grief are seen as insurmountable and can even lead to psychological illness. The process of social interaction also influences the way in which pregnancy losses are experienced, with fragility in healthcare and a lack of awareness among third parties being observed, which favors the extension of suffering and psychosocial illness, signaling challenges to be overcome by care services for women with SLE in the pregnancy-puerperal cycle.
